# Health literacy in Korea: findings from the 2023 Korea National Health and Nutrition Examination Survey

**DOI:** 10.4178/epih.e2025037

**Published:** 2025-07-09

**Authors:** Sunhye Choi, Yukyeong Kang, Hyejin Kim, Kyungwon Oh

**Affiliations:** Division of Health and Nutrition Survey and Analysis, Bureau of Chronic Disease Prevention and Control, Korea Disease Control and Prevention Agency, Cheongju, Korea

**Keywords:** Health literacy, Adult, Korea National Health and Nutrition Examination Survey

## Abstract

**OBJECTIVES:**

This study aimed to assess health literacy and identify vulnerable groups, providing a basis for developing health policies aimed at improving health literacy, using data from the 2023 Korea National Health and Nutrition Examination Survey (KNHANES).

**METHODS:**

The health literacy measurement tool used in the 2023 KNHANES comprised a total of 10 items spanning the domains of disease prevention, health promotion, healthcare, and technology and resources. Health literacy was analyzed in relation to socio-demographic characteristics and major health behaviors among 5,906 adults aged 19 years or older, using the SAS program.

**RESULTS:**

As of 2023, the overall prevalence of adequate health literacy among adults was 60.4%. Adequate health literacy was higher in women (62.2%) than in men (58.6%). Younger individuals exhibited higher levels of health literacy, with those aged 19–64 years at 65.9%, compared to only 40.3% among those aged 65 or older. Higher income and education levels were also associated with greater health literacy. Regarding health behavior characteristics, individuals practicing healthy lifestyles, such as non-smoking, engaging in physical activity, and undergoing health checkups, demonstrated higher health literacy than those who did not engage in such behaviors.

**CONCLUSIONS:**

Six out of 10 Korean adults demonstrated adequate health literacy, but significant differences were observed according to socio-demographic characteristics (e.g., age and education) and health behaviors (e.g., smoking and physical activity). Tailored education and policy initiatives are necessary to improve health literacy, particularly targeting older adults, low-income groups, individuals with lower education, and those who do not practice healthy lifestyles.

## GRAPHICAL ABSTRACT

[Fig f1-epih-47-e2025037]

## Key Message

According to the results of the 2023 KNHANES—the first nationwide assessment of health literacy in Korea—6 out of 10 Korean adults had adequate health literacy. Diverse strategies—including targeted education and information provision— are needed to improve health literacy among vulnerable groups such as older adults, those with low education and individuals who do not practice healthy lifestyles.

## INTRODUCTION

Recently, with the expansion of the internet and various media, individuals are exposed to vast amounts of health information; however, the expertise and reliability of this information are not always assured. This situation requires individuals not only to access health information, but also to select appropriate information, understand it, and utilize it effectively. The World Health Organization (WHO) defines health literacy as the ability to access, understand, evaluate, and use information and services to maintain and promote good health and well-being [1]. Health literacy encompasses more than simply accessing websites, reading pamphlets, or following prescribed health promotion behaviors; it also involves the ability to think critically, interact with others, and express both personal and social health needs [1]. From a public health perspective, health literacy is recognized as a key health determinant, and its importance continues to grow due to its strong relationship with health equity [2].

To improve health literacy, interventions are needed not only at the individual level, but also at the organizational, community, and governmental levels, with particular attention to health inequalities among marginalized and vulnerable groups. Accordingly, international organizations and numerous countries have made improving health literacy a central health policy agenda, and have undertaken continuous efforts to raise national health literacy. The WHO, for example, designated health literacy as one of the key strategies for health promotion through the Shanghai Declaration in 2016 [4]. The United States established a national action plan for improving health literacy in 2010, and the Healthy People initiative is advancing equity in health literacy through integrated policy goals and implementation strategies, emphasizing organizational responsibility from a public health standpoint [5,6]. Similarly, the United Kingdom has prioritized health literacy improvement as an important means to achieve health equity, with each local government formulating its own action plan and strategies [7,8]. Canada has also enacted policies to address inequalities arising from health literacy disparities and has proposed targeted solutions [9].

In Korea, the Korea Disease Control and Prevention Agency (KDCA) has been providing verified health information nationwide by operating the National Health Information Portal since 2017 [10]. The Ministry of Health and Welfare has also set specific policy directions, including periodic monitoring and the establishment of a health literacy education system, by adding “adequate level of health literacy” as a leading health indicator to the 5th National Health Plan (HP2030) to promote health equity through improved understanding and use of health information. “Adequate level of health literacy” was introduced for the first time as leading health indicators in HP2030 [11]. To enable regular monitoring of domestic health literacy, the KDCA developed and validated a health literacy measurement tool in 2022, which was subsequently incorporated into the KNHANES, Korea’s national health survey representing the entire population [12]. Health literacy surveys for adults in KNHANES have been conducted since 2023.

This study assessed the adequate health literacy of the Korean adult population and identified factors associated with health literacy, including socio-demographic characteristics and major health behaviors.

## MATERIALS AND METHODS

### Study population

The KNHANES is a national health survey conducted annually by the KDCA since 1998 to estimate the health and nutritional status of the Korean population. The KNHANES is designed to collect health information from the general public, targeting approximately 10,000 household members aged 1 year or older in 4,800 households across 192 survey districts nationwide. The survey sample is selected using a 2-stage stratified cluster sampling method, with survey districts and households serving as the first and second sampling units, respectively. The primary extraction frame is the Population and Housing Census data, and stratification variables include city/province, region (*dong, eup/myeon*), and housing type (general/apartment). Additionally, the ratio of residential area, age of household head, and ratio of single-person households are considered intrinsic stratification variables. In 2023, the KNHANES participation rate was 70.5%, and the number of participants aged 19 years or older was 5,906.

### Health literacy

The health literacy measurement tool used in KNHANES, developed through the KDCA policy research project in 2022, consists of 10 self-reported items (3 items on disease prevention, 1 on health promotion, 4 on healthcare, and 2 on technology and resources), rated on a 4-point Likert scale (never: 1 point, rarely: 2 points, sometimes: 3 points, and always: 4 points). Detailed information on the development and validity of the tool is available elsewhere [12]. Health literacy was assessed among adults aged 19 years or older using a self-administered internet or paper questionnaire. The initial cut-off score for adequate health literacy was defined as the percentage of individuals scoring 28 or higher (out of a maximum of 40) on the 10-item scale [13]. However, this threshold—derived from a small-scale pilot survey—had limitations, prompting the need for adjustment based on the results of the 2023 KNHANES, a representative survey of the Korean population. Following a review by the national health survey advisory committee, the KDCA raised the cut-off score to 30 or higher, taking into account factors such as high specificity, the risk of overestimating health literacy in self-reported measures, and other methodological considerations. The committee recommended that screening tools maintain a sensitivity of at least 70% and a specificity of at least 80%. A score of 28 or higher corresponded to a correct-answer rate of 67% for all adults and 35% for those aged 65 or older on knowledge-based items; raising the threshold to 30 or higher increased these rates to about 70% and 40%, respectively. Additionally, if any of the 10 self-reported items were marked as “don’t know” or “no response,” a score of 1 (corresponding to ‘never’) was assigned for those items when calculating the total score.

### Socio-demographic characteristics and health-related behaviors

Socio-demographic characteristics were categorized by gender (men, women); age (19-29, 30-39, 40-49, 50-59, 60-69, 70 or older, 19-64, 65 or older); residential area (*dong, eup/myeon*); household income (low, lower middle, middle, upper middle, high); marital status (married, divorced/widowed/other, unmarried); education level; economic activity; and occupational group (manual labor, non-manual labor). For education level, those aged 19-64 were grouped as high school graduate or lower versus college graduate or higher, and those 65 or older as middle school graduate or lower versus high school graduate or higher. Economic activity and occupational group were analyzed only for participants aged 19-64. Chronic disease was defined as having at least 1 of hypertension, diabetes, dyslipidemia, or obesity. Health care utilization was categorized as having used outpatient services in the past 2 weeks or having been hospitalized in the past year. Unmet medical need was defined as having experienced unmet medical need (hospital/clinic) in the past year. Health checkups were classified as having undergone a checkup in the past 2 years, and influenza vaccination as having received a vaccination in the past year. Smoking status was defined as current use of conventional cigarettes, and drinking as having consumed alcohol at least once a month in the past year. Physical activity was defined as engaging in moderate-intensity physical activity for at least 2 hours and 30 minutes per week, vigorous-intensity physical activity for at least 1 hour and 15 minutes per week, or a combination of both (with 1 minute of vigorous intensity equating to 2 minutes of moderate intensity) for an equivalent total amount. Depression experience were identified as feelings of sadness or despair that interfered with daily life for more than 2 consecutive weeks in the past year. Subjective health perception was categorized as “very good/good,” “fair,” or “bad/very bad.”

### Statistical analysis

Data analysis was conducted using SAS version 9.4 (SAS Institute Inc., Cary, NC, USA), with all results calculated using complex sample design analysis methods and weighted to represent the Korean population. Adequate health literacy in KNHANES was calculated using only 2023 data, and crude rates were reported for gender, age, residential area, income level, and education level. Statistical analyses were performed using SAS procedures (PROC SURVEYMEANS, PROC SURVEYLOGISTIC). To identify factors associated with health literacy, socio-demographic and health behavior variables that were statistically significant were selected, and multivariate regression analysis was performed. Statistical significance was determined at the 0.05 level.

## RESULTS

### Socio-demographic characteristics

The socio-demographic characteristics of 5,906 adults aged 19 years or older who participated in the 2023 KNHANES—including age, residential area, and income level—were analyzed, and the distribution by gender is presented in Table 1.

### Health literacy scores by domains

The health literacy measurement tool comprises 4 domains: disease prevention, health promotion, healthcare, and technology and resources. Responses are recorded on a 4-point Likert scale. The distribution of responses and mean scores for each item were analyzed (Table 2). In the disease prevention domain, which contains 3 items, the overall mean was 2.9 points (item 1: 2.8, item 2: 2.8, item 3: 2.9). The health promotion domain, represented by a single item, had a mean of 3.0 points. The healthcare domain, consisting of 4 items, had an overall mean of 3.1 points (item 5: 3.1, item 6: 2.9, item 7: 3.2, item 8: 3.1). The technology and resources domain, with 2 items, had an overall mean of 2.9 points (item 9: 2.8, item 10: 2.9). Among all domains, healthcare had the highest mean score, followed by health promotion, disease prevention, and technology and resources. The overall mean score for the 10 health literacy items, out of a total of 40 possible points, was 29.7 points. The average scores for men (29.6 points) and women (29.7 points) were similar.

### Health literacy by socio-demographic characteristics

The proportion of adults with adequate health literacy (scoring 30 points or more out of 40) was 60.4% overall. Among women, 62.2% met the adequate threshold compared to 58.6% of men. In the 19-64 age group, women (70.5%) had higher health literacy than men (61.6%), while among those aged 65 or older, men (45.8%) had higher scores than women (35.9%). For both genders, health literacy was higher among younger participants: those in their 20s had the highest rate (70.5%), whereas those aged 70 or older had the lowest (36.0%). Regionally, health literacy was higher in the *dong* area (62.3%) than in the *eup/myeon* area (51.0%), and it increased with income level. The difference between the ‘high’ (66.3%) and ‘low’ (54.4%) income groups was 11.9 percentage points (%p), with a larger gap for men (14.7%p) than for women (9.1%p). Health literacy was also higher among those with higher educational attainment (aged 19-64: high school graduate or lower, 57.0%; college graduate or higher, 71.9%; aged 65 or older: middle school graduate or lower, 32.2%; high school graduate or higher, 59.7%). For those aged 19-64, there was no significant difference in health literacy according to economic activity, but by occupational group, non-manual laborers (71.8%) had higher literacy than manual laborers (59.7%) (Table 3).

### Health literacy by health behaviors

Non-smokers (64.3%) exhibited higher health literacy than current smokers (53.2%), and those who engaged in adequate physical activity (65.4%) had higher health literacy than those who did not (59.6%). Individuals without depression experience (63.4%) had higher health literacy than those with depression experience (52.9%), and those with a subjective health perception of “good or very good” (70.3%) scored higher than those reporting “bad or very bad” (59.6%) or “fair” (53.4%). Participants who did not experience unmet medical needs in the past year (62.8%) had higher health literacy than those who did (56.2%), and those who received a health checkup in the past 2 years (64.4%) scored higher than those who did not (56.8%). There were no statistically significant differences in health literacy with respect to alcohol drinking, chronic disease status, health care utilization, or influenza vaccination (Table 4).

### Health literacy and associated factors

To identify factors associated with health literacy, statistically significant variables from socio-demographic and health behavior characteristics were selected for multivariate regression analysis. Health literacy was higher among those aged 19-29 (adjusted odds ratio [aOR], 2.45) compared to those aged 70 or older, and among individuals with a college education or higher (aOR, 1.79) compared to those with a high school education or lower. Also higher income levels were associated with higher health literacy, this relationship was not statistically significant. Regarding health behaviors, non-smokers (aOR, 1.50) had higher health literacy than smokers; individuals practicing adequate physical activity (aOR, 1.19) scored higher than those with inadequate activity; and those without depression experience (aOR, 1.29) scored higher than those with depression experience. Among men, those without unmet medical needs (aOR, 1.51) were more likely to have higher health literacy; among women, those who had undergone health checkups (aOR, 1.23) were more likely to have higher health literacy than those who had not. Additionally, those who rated their subjective health as “good or very good” (aOR, 1.56) were more likely to have higher health literacy compared to those with a “bad or very bad” perception (Table 5).

## DISCUSSION

This is the first study to evaluate health literacy in a representative sample of the Korean population. The adequate health literacy rate among Korean adults was 60.4%, meaning that 6 out of 10 people had adequate health literacy. However, among adults aged 65 or older, only 4 out of 10 demonstrated adequate health literacy. In particular, health literacy was notably lower among the elderly, low-income groups, those with lower education levels, and individuals who did not practice healthy lifestyles compared to other groups. Additionally, an analysis of health literacy by domain revealed that scores for technology and resources were lower than those for health promotion and healthcare. Notably, the component in which the population was asked to judge whether the information they obtained was correct and then to utilize it was found to be especially weak.

Compared to previous domestic studies, the health literacy of people aged 19-69, as measured by the Health Literacy Survey European Union Questionnaire 16 tool (HLS-EU-Q16) by the Korea Institute for Health and Social Affairs, was found to be inadequate (0-8 points) in 43.4% of respondents, and adequate (13-16 points) in 29.1%. The mean score was 9.1 points out of 16 points, with mean scores higher among women than men, married than single individuals, those living in large cities, those with higher subjective social class, and those living in households of 2 or more people. Health literacy, as measured by health behaviors, was lower in cases of poor subjective health status, lack of physical activity, and experience of unmet medical needs [3]. The mean health literacy score surveyed by the Korea Health Panel Survey using the HLS-EU-Q16 was 11.3 points, with 50.6% of respondents achieving adequate health literacy (13-16 points) and 29.3% at an inadequate level (0-8 points). The mean was lower for women than men, for those in older age groups, for those with lower education and income levels, for those not economically active, and for those living in *eup/myeon* areas compared to *dong* areas [14]. Most previous studies on domestic health literacy were conducted among small, specific population groups, with only a few studies examining representative samples as noted above. Furthermore, measurement tools and domains varied, making direct comparison with this study’s results difficult. Nevertheless, the factors associated with health literacy were similar, and prior research likewise reported that while access to and understanding of health information was generally not problematic, there was some difficulty in evaluating information, consistent with the findings of this study [15].

In an international study conducted in 8 European countries using the HLS-EU measurement tool, 1 in 10 people demonstrated inadequate health literacy, 1 in 2 people had limited (inadequate or problematic) health literacy, and there were differences in health literacy levels between countries (29-62%) [16]. These results mirrored those in Korea, with limited health literacy more prevalent among those with financial deprivation, low social status, low education, and older age. In the United States, improving health literacy has been established as a social determinant of health in Healthy People 2020 and 2030, and is monitored as an objective within the health communication and health information technology domains [17]. Among the main objectives, 8.9% of adults in 2021 reported inadequate communication with medical service providers, slightly missing the target of 8% [18].

In Korea, the HP2030 objective for improving health literacy sets targets for adequate health literacy at 70% for adults and 50% for the elderly, but the results in 2023 fell short of these targets. Health literacy by age group showed that women aged 19-64 had higher literacy than men, while among those 65 or older, men had higher literacy than women. As health literacy patterns differ by gender across age groups, further research is needed to understand the underlying socio-cultural factors, and efforts should be tailored accordingly. With respect to income quintiles, there was a greater disparity among men (14.7%p) than among women (9.1%p), indicating particular vulnerability among low-income men. Consistent with the study’s findings, health literacy was low among men, those over 65 years of age, individuals with lower education levels, and those with lower incomes, emphasizing the need for multifaceted interventions to improve health literacy among vulnerable groups. Additionally, differences in health literacy by residential area suggest that local governments should implement strategies to address health literacy in regional health promotion plans. Other studies have also reported that parental health literacy is closely linked to adolescent health literacy [20], and that health literacy is low among domestic immigrants, such as married immigrant women, foreign workers, and North Korean defectors [19]. Further research, policy development, and programs are needed to both achieve adequate health literacy and to eliminate health disparities and improve health outcomes among vulnerable populations. Moreover, the ability to judge and utilize information was lower than scores for health promotion and healthcare. As internet and smart device use becomes increasingly widespread, education and promotion for proper information use should be prioritized, with close cooperation and ongoing support among government, local authorities, and educational institutions.

A limitation of this study is that health literacy was surveyed via self-reported questionnaires, so there is a possibility of overestimating participants’ ability to understand health information. However, both self-reported and knowledge-based items were included in this study, and the concordance rate between those scoring 30 or higher on self-reported items and those correctly answering the knowledge-based item was approximately 68.9%. We plan to periodically include knowledge-based items in future surveys to evaluate the level and nature of discrepancies between knowledge-based and self-reported responses.

In conclusion, 6 out of 10 Korean adults had adequate health literacy according to the first nationwide assessment of health literacy in Korea. However, health literacy decreases among older adults, individuals with low education and income, and those who do not engage in healthy lifestyles. To address these disparities, diverse strategies—including targeted education and tailored information provision—are required to improve health literacy among these vulnerable populations.

## Figures and Tables

**Figure f1-epih-47-e2025037:**
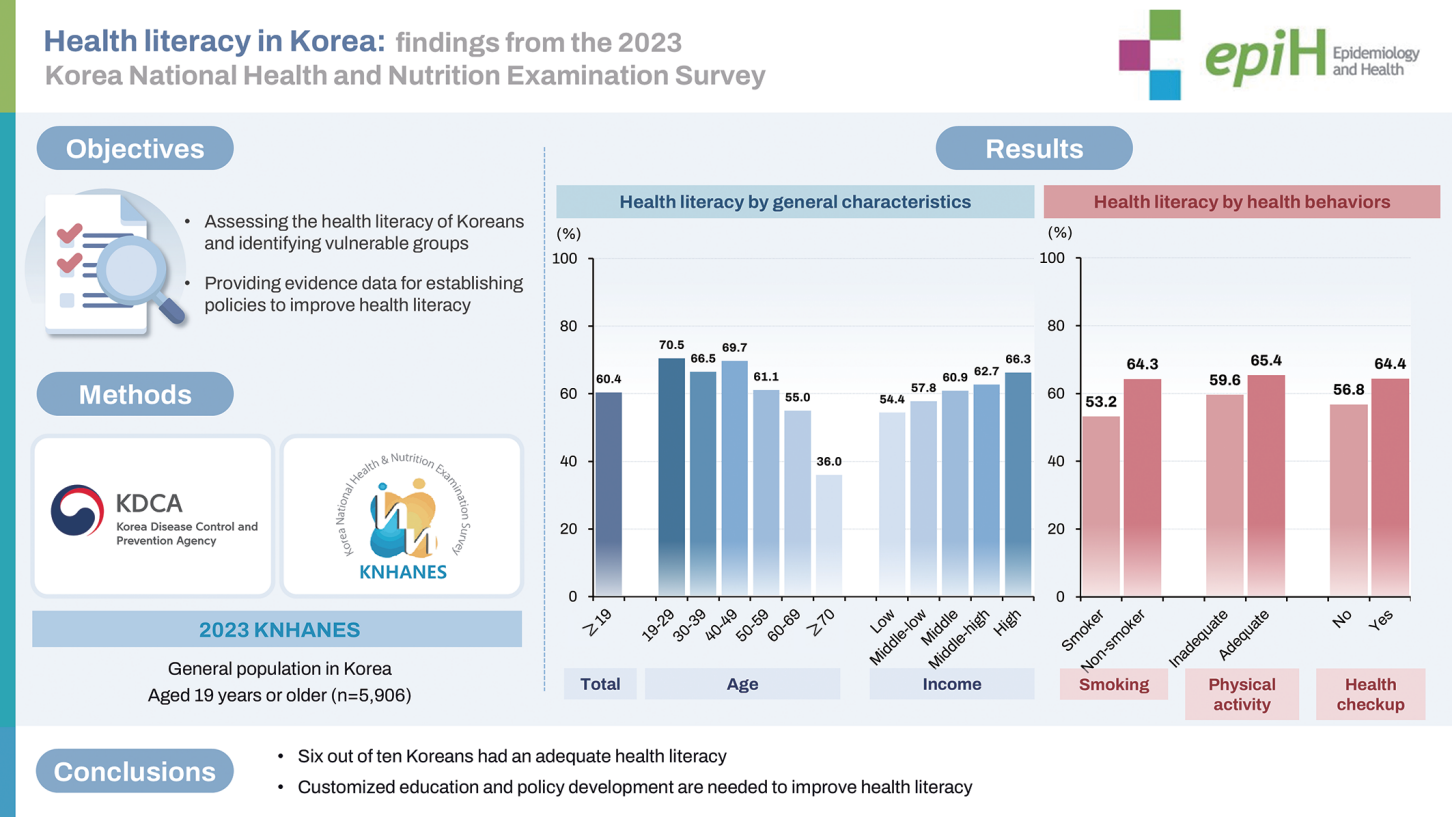


**Table 1.** Socio-demographic characteristics among Korean men and women aged 19 years or older, the 2023 Korea National Health and Nutrition Examination Survey

Characteristics	Total		Men		Women	
Total	5,906	100 (0.0)	2,573	49.7 (0.6)	3,333	50.3 (0.6)
Age (yr)						
19-29	611	15.7 (0.7)	287	16.5 (0.9)	324	14.8 (0.9)
30-39	679	15.6 (0.9)	304	16.6 (1.1)	375	14.6 (0.9)
40-49	981	18.1 (0.8)	428	18.7 (1.0)	553	17.6 (0.9)
50-59	1,113	19.5 (0.6)	446	19.7 (0.9)	667	19.3 (0.8)
60-69	1,336	17.1 (0.7)	580	16.8 (0.9)	756	17.4 (0.7)
≥70	1,186	14.0 (0.7)	528	11.7 (0.7)	658	16.2 (1.0)
Region						
Dong	4,709	83.4 (2.6)	2,022	82.2 (2.8)	2,687	84.6 (2.5)
*Eup/Myeon*	1,197	16.6 (2.6)	551	17.8 (2.8)	646	15.4 (2.5)
Income						
Low	1,175	19.5 (0.9)	509	19.3 (1.1)	666	19.6 (1.0)
Middle-low	1,176	20.2 (0.8)	515	20.0 (1.0)	661	20.4 (1.0)
Middle	1,180	20.1 (0.8)	515	20.4 (1.1)	665	19.8 (0.9)
Middle-high	1,174	20.1 (0.8)	513	20.6 (1.1)	661	19.7 (0.9)
High	1,176	20.1 (1.2)	512	19.7 (1.3)	664	20.5 (1.3)
Education						
≤Middle school	1,410	17.6 (1.0)	482	12.4 (0.9)	928	22.8 (1.2)
≤High school	1,607	27.1 (0.9)	701	26.7 (1.1)	906	27.6 (1.1)
≥College	2,737	55.2 (1.5)	1,330	60.9 (1.6)	1,407	49.7 (1.6)
Economic activity						
Yes	3,341	60.5 (0.9)	1,656	68.6 (1.2)	1,685	52.4 (1.1)
No	2,565	39.5 (0.9)	917	31.4 (1.2)	1,648	47.6 (1.1)

Values are presented as number or % (standard error).

**Table 2.** Health literacy scores by domains among Korean men and women aged 19 years or older, the 2023 Korea National Health and Nutrition Examination Survey

Domains	Items	Mean (points) (SE)	Never (1 point)	Rarely (2 points)	Sometimes (3 points)	Always (4 points)	Don’t know/no response (1 point)
Total							
Disease prevention	1. Can you assess which vaccinations are necessary?	2.8 (0.0)	7.0 (0.5)	17.0 (0.6)	56.2 (0.7)	17.2 (0.6)	2.6 (0.3)
	2. Do you understand the degree of risk of mental health issues such as stress and depression?	2.8 (0.0)	7.4 (0.5)	13.4 (0.5)	59.0 (0.8)	17.7 (0.7)	2.6 (0.3)
	3. Are you aware of the health symptoms resulting from excessive drinking, smoking, and lack of exercise?	2.9 (0.0)	5.1 (0.4)	9.9 (0.5)	62.5 (0.8)	19.9 (0.7)	2.7 (0.3)
	Overall (mean)	2.9 (0.0)					
Health promotion	4. Can you evaluate how daily behaviors influence health?	3.0 (0.0)	4.3 (0.4)	8.5 (0.4)	66.9 (0.7)	17.7 (0.7)	2.7 (0.3)
Healthcare	5. Do you have difficulty understanding explanations and instructions from doctors during medical appointments?	3.1 (0.0)	2.0 (0.2)	3.3 (0.2)	65.1 (0.8)	27.1 (0.7)	2.5 (0.2)
	6. Can you assess what needs to be prioritized in case of an emergency?	2.9 (0.0)	3.1 (0.3)	11.1 (0.5)	66.5 (0.8)	16.8 (0.6)	2.5 (0.3)
	7. Do you understand how to take medications as explained by doctors or pharmacists?	3.2 (0.0)	1.4 (0.2)	2.1 (0.2)	61.6 (0.9)	32.5 (0.9)	2.4 (0.2)
	8. Do you understand patient education materials provided by hospitals?	3.1 (0.0)	2.7 (0.3)	6.4 (0.4)	62.2 (0.8)	26.2 (0.8)	2.6 (0.3)
	Overall (mean)	3.1 (0.1)					
Technology and resources	9. Can you assess the reliability of health information obtained from the internet or media (e.g., television, YouTube)?	2.8 (0.0)	4.3 (0.3)	16.5 (0.6)	62.1 (0.8)	14.4 (0.6)	2.6 (0.3)
	10. Can you apply health information obtained from the internet or media (e.g., television, YouTube) to actual health-related behaviors or decision making?	2.9 (0.0)	4.5 (0.3)	14.1 (0.6)	64.6 (0.8)	14.2 (0.6)	2.6 (0.3)
	Overall (mean)	2.9 (0.0)					
Total score (mean)		29.7 (0.1)					
Men							
Disease prevention	1. Can you assess which vaccinations are necessary?	2.8 (0.0)	8.3 (0.7)	19.4 (0.9)	54.6 (1.1)	15.7 (0.8)	2.1 (0.3)
	2. Do you understand the degree of risk of mental health issues such as stress and depression?	2.8 (0.0)	8.4 (0.7)	15.3 (0.8)	57.5 (1.1)	16.6 (0.9)	2.2 (0.3)
	3. Are you aware of the health symptoms resulting from excessive drinking, smoking, and lack of exercise?	2.9 (0.0)	5.0 (0.5)	11.0 (0.8)	62.7 (1.2)	19.2 (0.9)	2.2 (0.3)
	Overall (mean)	2.8 (0.1)					
Health promotion	4. Can you evaluate how daily behaviors influence health?	2.9 (0.0)	4.3 (0.5)	8.7 (0.6)	68.1 (1.1)	16.7 (0.9)	2.2 (0.3)
Healthcare	5. Do you have difficulty understanding explanations and instructions from doctors during medical appointments?	3.1 (0.0)	2.0 (0.3)	3.2 (0.4)	67.8 (1.1)	25.0 (1.0)	2.0 (0.3)
	6. Can you assess what needs to be prioritized in case of an emergency?	3.0 (0.0)	2.8 (0.4)	9.9 (0.6)	68.2 (1.1)	17.1 (0.8)	2.0 (0.3)
	7. Do you understand how to take medications as explained by doctors or pharmacists?	3.2 (0.0)	1.4 (0.3)	2.3 (0.3)	64.6 (1.2)	29.7 (1.2)	2.0 (0.3)
	8. Do you understand patient education materials provided by hospitals?	3.1 (0.0)	2.1 (0.3)	5.6 (0.5)	65.9 (1.1)	24.2 (1.1)	2.1 (0.3)
	Overall (mean)	3.1 (0.1)					
Technology and resources	9. Can you assess the reliability of health information obtained from the internet or media (e.g., television, YouTube)?	2.9 (0.0)	3.8 (0.4)	16.9 (0.7)	63.0 (1.1)	14.2 (0.8)	2.1 (0.3)
	10. Can you apply health information obtained from the internet or media (e.g., television, YouTube) to actual health-related behaviors or decision making?	2.9 (0.0)	4.0 (0.4)	14.9 (0.9)	65.4 (1.2)	13.6 (0.9)	2.1 (0.3)
	Overall (mean)	2.9 (0.0)					
Total score (mean)		29.6 (0.1)					
Women							
Disease prevention	1. Can you assess which vaccinations are necessary?	2.9 (0.0)	5.7 (0.5)	14.6 (0.7)	57.9 (0.8)	18.8 (0.8)	3.0 (0.4)
	2. Do you understand the degree of risk of mental health issues such as stress and depression?	2.9 (0.0)	6.3 (0.5)	11.4 (0.7)	60.5 (1.1)	18.9 (0.9)	2.9 (0.4)
	3. Are you aware of the health symptoms resulting from excessive drinking, smoking, and lack of exercise?	2.9 (0.0)	5.1 (0.4)	8.8 (0.6)	62.3 (1.0)	20.5 (0.9)	3.3 (0.4)
	Overall (mean)	2.9 (0.1)					
Health promotion	4. Can you evaluate how daily behaviors influence health?	3.0 (0.0)	4.2 (0.4)	8.2 (0.6)	65.6 (1.0)	18.8 (0.9)	3.1 (0.4)
Healthcare	5. Do you have difficulty understanding explanations and instructions from doctors during medical appointments?	3.2 (0.0)	2.0 (0.3)	3.4 (0.3)	62.5 (1.0)	29.2 (1.0)	2.9 (0.3)
	6. Can you assess what needs to be prioritized in case of an emergency?	2.9 (0.0)	3.4 (0.4)	12.3 (0.6)	64.8 (0.9)	16.5 (0.7)	3.0 (0.4)
	7. Do you understand how to take medications as explained by doctors or pharmacists?	3.2 (0.0)	1.4 (0.3)	2.0 (0.3)	58.6 (1.1)	35.2 (1.1)	2.9 (0.3)
	8. Do you understand patient education materials provided by hospitals?	3.1 (0.0)	3.2 (0.4)	7.1 (0.5)	58.5 (0.9)	28.1 (1.0)	3.0 (0.4)
	Overall (mean)	3.1 (0.1)					
Technology and resources	9. Can you assess the reliability of health information obtained from the internet or media (e.g., television, YouTube)?	2.8 (0.0)	4.8 (0.4)	16.2 (0.8)	61.3 (1.0)	14.7 (0.7)	3.2 (0.4)
	10. Can you apply health information obtained from the internet or media (e.g., television, YouTube) to actual health-related behaviors or decision making?	2.9 (0.0)	5.0 (0.4)	13.3 (0.6)	63.9 (1.0)	14.7 (0.7)	3.2 (0.4)
	Overall (mean)	2.9 (0.0)					
Total score (mean)		29.7 (0.2)					

Values are presented as % (SE). SE, standard error.

**Table 3.** Health literacy^1^ by socio-demographic characteristics among Korean men and women aged 19 years or older, the 2023 Korea National Health and Nutrition Examination Survey

Characteristics	Total		p-value	Men		p-value	Women		p-value
Total	5,906	60.4 (1.0)		2,573	58.6 (1.1)		3,333	62.2 (1.2)	
Age (yr)									
19-29	611	70.5 (2.2)	<0.001	287	66.7 (3.3)	<0.001	324	74.7 (2.5)	<0.001
30-39	679	66.5 (1.7)		304	61.5 (2.6)		375	72.1 (2.3)	
40-49	981	69.7 (1.6)		428	64.3 (2.3)		553	75.3 (2.1)	
50-59	1,113	61.1 (1.6)		446	56.2 (2.3)		667	66.0 (1.9)	
60-69	1,336	55.0 (1.8)		580	55.8 (2.4)		756	54.3 (2.2)	
≥70	1,186	36.0 (1.9)		528	42.1 (2.4)		658	31.7 (2.2)	
19-64	4,070	65.9 (0.9)	<0.001	1,761	61.6 (1.2)	<0.001	2,309	70.5 (1.1)	<0.001
≥65	1,836	40.3 (1.6)		812	45.8 (2.1)		1,024	35.9 (1.9)	
Region			0.029			0.224			0.010
Dong	4,709	62.3 (1.0)		2,022	60.0 (1.2)		2,687	64.5 (1.2)	
*Eup/Myeon*	1,197	51.0 (3.3)		551	52.3 (3.7)		646	49.5 (3.5)	
Income^2^			<0.001			<0.001			<0.001
Low	1,175	54.4 (1.9)		509	51.3 (2.7)		666	57.4 (2.6)	
Middle-low	1,176	57.8 (1.8)		515	56.1 (2.7)		661	59.4 (2.1)	
Middle	1,180	60.9 (2.1)		515	59.7 (2.8)		665	62.2 (2.3)	
Middle-high	1,174	62.7 (1.6)		513	59.7 (2.4)		661	65.7 (2.1)	
High	1,176	66.3 (1.5)		512	66.0 (2.2)		664	66.5 (1.9)	
Marital status			<0.001			<0.001			<0.001
Married	3,953	62.1 (1.0)		1,798	59.1 (1.3)		2,155	65.2 (1.3)	
Divorce, death	877	41.6 (2.1)		207	46.0 (3.9)		670	40.0 (2.2)	
Unmarried	1,072	65.0 (1.6)		567	60.2 (2.4)		505	71.8 (2.1)	
Education									
19-64 yr			<0.001			<0.001			<0.001
≤High school	1,496	57.0 (1.6)		553	50.0 (2.5)		943	62.8 (1.9)	
≥College	2,515	71.9 (1.0)		1,180	68.0 (1.4)		1,335	76.5 (1.2)	
≥65 yr			<0.001			<0.001			<0.001
≤Middle school	1,103	32.2 (1.7)		388	35.9 (2.9)		715	30.2 (2.0)	
≥High school	640	59.7 (2.1)		392	58.4 (2.7)		248	62.0 (3.3)	
Economic activity									
19-64 yr			0.409			0.646			0.703
Yes	2,705	65.7 (1.0)		1,328	62.2 (1.4)		1,377	70.3 (1.3)	
No	1,365	66.4 (1.5)		433	59.8 (2.8)		932	70.6 (1.7)	
Occupation^3^									
19-64 yr			<0.001			<0.001			<0.001
Manual	1,389	59.7 (1.4)		706	56.3 (2.0)		683	64.6 (1.8)	
Non-manual	1,316	71.8 (1.3)		622	68.6 (1.9)		694	75.7 (1.7)	

Values are presented as number or % (standard error).

1 The appropriate level of health literacy was defined as the percentage of people with a score of 30 or higher (on a scale of 1 to 4) based on the total score of 10 self-reported items.

2 Monthly equivalized household income (monthly household income/√number of household members) is classified into quintiles by gender and age (in 5-year units).

3 Non-manual (Korean Standard Classification of Occupations [KSCO] 1,2,3), manual (KSCO 4,5,6,7,8,9,10).

**Table 4.** Health literacy^1^ by health behaviors among Korean men and women aged 19 years or older, the 2023 Korea National Health and Nutrition Examination Survey

Variables	Total		p-value	Men		p-value	Women		p-value
Smoking			<0.001			<0.001			0.042
Smoker	908	53.2 (2.0)		764	53.5 (2.1)		144	54.2 (5.3)	
Non-smoker	4,855	64.3 (0.9)		1,751	63.1 (1.4)		3,104	65.3 (1.1)	
Alcohol drinking			0.496			0.608			0.378
Drinker	2,947	62.6 (1.1)		1,647	60.3 (1.4)		1,300	65.7 (1.5)	
Non-drinker	2,825	61.6 (1.2)		874	59.1 (2.0)		1,951	64.0 (1.4)	
Physical activity			<0.001			0.014			0.002
Inadequate	2,929	59.6 (1.2)		1,206	56.8 (1.7)		1,723	62.3 (1.5)	
Adequate	2,459	65.4 (1.2)		1,121	63.1 (1.8)		1,338	68.1 (1.4)	
Depression experience			<0.001			0.022			<0.001
Yes	644	52.9 (2.5)		214	51.6 (3.8)		430	52.9 (2.6)	
No	5,123	63.4 (0.9)		2,304	60.7 (1.2)		2,819	66.6 (1.2)	
Chronic disease			0.090			0.463			0.670
Yes	3,456	60.8 (1.1)		1,628	59.2 (1.4)		1,828	63.8 (1.4)	
No	1,909	63.8 (1.4)		712	61.1 (2.1)		1,197	64.8 (1.8)	
Health care utilization			0.507			0.356			0.650
Yes	1,923	61.5 (1.4)		783	58.4 (2.2)		1,140	64.2 (1.8)	
No	3,837	62.6 (1.0)		1,732	60.7 (1.3)		2,105	65.1 (1.3)	
Unmet medical need			0.010			0.007			0.171
Yes	474	56.2 (2.5)		165	49.6 (4.1)		309	60.5 (3.3)	
No	5,186	62.8 (0.9)		2,286	60.7 (1.2)		2,900	65.2 (1.1)	
Health checkup			<0.001			0.071			<0.001
No	1,405	56.8 (1.8)		632	56.0 (2.6)		773	58.6 (2.1)	
Yes	3,996	64.4 (1.0)		1,699	61.4 (1.3)		2,297	67.2 (1.2)	
Influenza vaccination			0.076			0.104			0.904
No	2,887	61.0 (1.0)		1,356	58.4 (1.5)		1,531	64.7 (1.4)	
Yes	2,873	63.8 (1.3)		1,158	62.5 (1.9)		1,715	64.9 (1.6)	
Subjective health perception			<0.001			<0.001			0.002
Bad/Very bad	2,607	59.6 (1.2)		1,087	54.9 (1.5)		1,520	64.3 (1.4)	
Fair	1,044	53.4 (1.7)		386	53.1 (2.8)		658	53.9 (2.2)	
Good/Very good	1,768	70.3 (1.3)		868	68.3 (1.7)		900	73.2 (1.8)	

Values are presented as number or % (standard error).

1 The appropriate level of health literacy was defined as the percentage of people with a score of 30 or higher (on a scale of 1 to 4) based on the total score of 10 self-reported items; Age and income level were adjusted and calculated during the analysis.

**Table 5.** Health literacy^1^ and associated factors among Korean men and women aged 19 years or older, the 2023 Korea National Health and Nutrition Examination Survey

Variables	Total	Men	Women
Age (yr)			
19-29	2.45 (1.88, 3.20)	1.55 (1.06, 2.26)	3.42 (2.26, 5.17)
30-39	1.85 (1.45, 2.36)	1.03 (0.73, 1.46)	3.14 (2.10, 4.69)
40-49	2.56 (2.03, 3.25)	1.54 (1.10, 2.16)	3.85 (2.70, 5.51)
50-59	2.09 (1.71, 2.56)	1.41 (1.04, 1.91)	2.76 (2.12, 3.60)
60-69	1.73 (1.41, 2.11)	1.40 (1.05, 1.86)	1.97 (1.53, 2.54)
≥70	1.00 (reference)	1.00 (reference)	1.00 (reference)
Income^2^			
Low	1.00 (reference)	1.00 (reference)	1.00 (reference)
Middle-low	0.99 (0.80, 1.23)	1.05 (0.77, 1.44)	0.96 (0.73, 1.27)
Middle	1.12 (0.89, 1.41)	1.17 (0.84, 1.64)	1.13 (0.85, 1.50)
Middle-high	1.15 (0.94, 1.41)	1.15 (0.85, 1.55)	1.23 (0.94, 1.59)
High	1.20 (0.95, 1.51)	1.24 (0.89, 1.74)	1.18 (0.87, 1.61)
Education			
≤High school	1.00 (reference)	1.00 (reference)	1.00 (reference)
≥College	1.79 (1.51, 2.12)	1.99 (1.53, 2.58)	1.68 (1.32, 2.15)
Smoking			
Smoker	1.00 (reference)	1.00 (reference)	1.00 (reference)
Non-smoker	1.50 (1.25, 1.79)	1.31 (1.05, 1.65)	1.05 (0.63, 1.72)
Physical activity			
Inadequate	1.00 (reference)	1.00 (reference)	1.00 (reference)
Adequate	1.19 (1.05, 1.36)	1.20 (0.97, 1.49)	1.19 (1.01, 1.40)
Depression experience			
Yes	1.00 (reference)	1.00 (reference)	1.00 (reference)
No	1.29 (1.02, 1.62)	1.27 (0.89, 1.82)	1.40 (1.08, 1.81)
Unmet medical need			
Yes	1.00 (reference)	1.00 (reference)	1.00 (reference)
No	1.18 (0.96, 1.46)	1.51 (1.07, 2.14)	1.06 (0.78, 1.44)
Health checkup			
No	1.00 (reference)	1.00 (reference)	1.00 (reference)
Yes	1.18 (0.99, 1.41)	1.13 (0.88, 1.45)	1.23 (1.01, 1.51)
Subjective health perception			
Bad/Very bad	1.00 (reference)	1.00 (reference)	1.00 (reference)
Fair	0.89 (0.75, 1.05)	1.05 (0.80, 1.36)	0.75 (0.61, 0.93)
Good/Very good	1.56 (1.33, 1.83)	1.65 (1.33, 2.05)	1.53 (1.23, 1.91)

Values are presented as adjusted odds ratio (95% confidence interval).

1 The appropriate level of health literacy was defined as the percentage of people with a score of 30 or higher (on a scale of 1 to 4) based on the total score of 10 self-reported items.

2 Monthly equivalized household income (monthly household income/√number of household members) is classified into quintiles by gender and age (in 5-year units).
